# Human arachnoid granulations Part I: a technique for quantifying area and distribution on the superior surface of the cerebral cortex

**DOI:** 10.1186/1743-8454-4-6

**Published:** 2007-07-16

**Authors:** Deborah M Grzybowski, Edward E Herderick, Kapil G Kapoor, David W Holman, Steven E Katz

**Affiliations:** 1 Department of Ophthalmology, Neuro Ophthalmology Research Division, The Ohio State University, 5^th ^Cramblett Hall, 456 West 10^th ^Avenue, Columbus, OH 43210, USA; 2 Biomedical Engineering Department, The Ohio State University, 270 Bevis Hall, 1080 Carmack Road, Columbus, OH 43210, USA; 3 eehscience, LLC, 31 Hill Road South, Pickerington, OH 43147, USA; 4 College of Medicine, The Ohio State University, 370 West 9th Avenue, Columbus, OH 43210, USA

## Abstract

**Background:**

The arachnoid granulations (AGs) are herniations of the arachnoid membrane into the dural venous sinuses on the surface of the brain. Previous morphological studies of AGs have been limited in scope and only one has mentioned surface area measurements. The purpose of this study was to investigate the topographic distribution of AGs on the superior surface of the cerebral cortex.

**Methods:**

*En face *images were taken of the superior surface of 35 formalin-fixed human brains. AGs were manually identified using Adobe Photoshop, with a pixel location containing an AG defined as 'positive'. A set of 25 standard fiducial points was marked on each hemisphere for a total of 50 points on each image. The points were connected on each hemisphere to create a segmented image. A standard template was created for each hemisphere by calculating the average position of the 25 fiducial points from all brains. Each segmented image was mapped to the standard template using a linear transformation. A topographic distribution map was produced by calculating the proportion of AG positive images at each pixel in the standard template. The AG surface area was calculated for each hemisphere and for the total brain superior surface. To adjust for different brain sizes, the proportional involvement of AGs was calculated by dividing the AG area by the total area.

**Results:**

The total brain average surface area of AGs was 78.53 ± 13.13 mm^2 ^(n = 35) and average AG proportional involvement was 57.71 × 10^-4 ^± 7.65 × 10^-4^. Regression analysis confirmed the reproducibility of AG identification between independent researchers with r^2 ^= 0.97. The surface AGs were localized in the parasagittal planes that coincide with the region of the lateral lacunae.

**Conclusion:**

The data obtained on the spatial distribution and *en face *surface area of AGs will be used in an *in vitro *model of CSF outflow. With an increase in the number of samples, this analysis technique can be used to study the relationship between AG surface area and variables such as age, race and gender.

## Background

The arachnoid granulations (AGs) are herniations of the arachnoid membrane that protrude through the dura mater and into the lateral lacunae and venous sinuses on the surface of the brain. They are associated primarily with drainage of cerebrospinal fluid (CSF) into the venous sinuses [1]. The AGs have been the focus of intensive study since Key and Retzius (1876) attributed the functional role of cerebrospinal fluid outflow to these "Pacchionian bodies" [2]. Most research over the last century has focused on further characterizing the ultrastructure and functional attributes of the AGs, with less attention to its macroscopic anatomy, distribution, and total surface area.

Little work has been done with respect to the *en face *surface area of the AGs on the superior surface of the cerebral cortex. In a morphological characterization of the CSF drainage pathway in the AGs of humans, Upton *et al*. offered some information on individual AG surface area [3]. Using light microscopy and SEM it was estimated that the apical cap was approximately 300 *μ*m in diameter and 150 *μ*m thick surmounting a collagenous core. This study used a sample size of 23 and offered little in terms of the methodology of these calculations or the demographics of the sample population. In addition, the surface area of individual AGs was calculated, without information on frequency or total surface area. It has been suggested that the actual number of AGs on the human brain vary with demographic factors [4]. Therefore, extrapolating individual AG data to whole brain AG surface area may not be accurate.

The earliest thorough account of the frequency and distribution of human AGs was made by Le Gros Clark [4]. He described the AGs as "most numerous in the floor of the lacunae laterales." He also described AGs as occurring directly in the superior sagittal sinus (SSS) "quite frequently". Le Gros Clark emphasized the distinction between the lateral lacunae and the sagittal sinus, regarding them as two separate anatomic entities, and stating that the lateral lacunae are not mere "diverticula of the sagittal sinus," but rather a complicated "meshwork of veins." He further described the position of the lateral lacunae as occurring alongside the SSS where there is "a coarse plexus formed by the anastomosis of the terminal arborizations of the meningeal veins." However, a recent study by Fox *et al*. noted that the lateral lacunae provide a direct pathway to the venous outflow system, which would allow for the outflow of CSF from the AGs to the venous system [5].

CSF outflow is now recognized as occurring through more than one pathway, with the arachnoid membrane, including both macroscopic AGs and villi (which we will define as being synonymous), in addition to the microvilli, playing an important role. This study addresses only the visible AGs, which may comprise only a portion of the potential outflow area within the arachnoid membrane, when compared to the microvilli. A separate future study is necessary to quantify the total outflow capability through the arachnoid membrane through microvilli in areas where no AGs are present. Even so, it is important to study and quantify the AGs independently because of their unique location, morphology, and structure [6].

The purpose of this study was to produce a topographic distribution map of human AG distribution on the superior surface of the cerebral cortex indicating the probability of an AG occurring in a particular location, in addition to calculation of total *en face *AG cap cell surface area. Photographic imaging of the superior surface of human brains was done to characterize AG distribution on the surface of the cerebral cortex, as well as the SSS.

## Methods

This study was performed in accordance with guidelines and regulations set forth by the Office of Responsible Research Practices Institutional Review Board for human subjects at The Ohio State University and The Declaration of Helsinki. Brains were first visually inspected after being formalin-fixed for 10 days in preparation for autopsy. All brains, independent of cause of death, were included in the study. This will allow for future analysis by disease state when the number of cases is increased. If the dura was still attached, the SSS was cut open and visually inspected for AGs and photographed. The dura was then carefully removed, ensuring that the arachnoid membrane remained intact in all locations. The dura was visually inspected for any signs of AGs and photographed. If a tear was observed in the arachnoid membrane after removal of the dura, the brain was excluded from the study. In most cases the dura remained in the skull-cap, therefore the SSS was not cut open while still attached to the brain. Removing the dura from the skull-cap frequently damaged the integrity of the dura. In these cases the SSS was excluded from the study.

**Figure 1:**
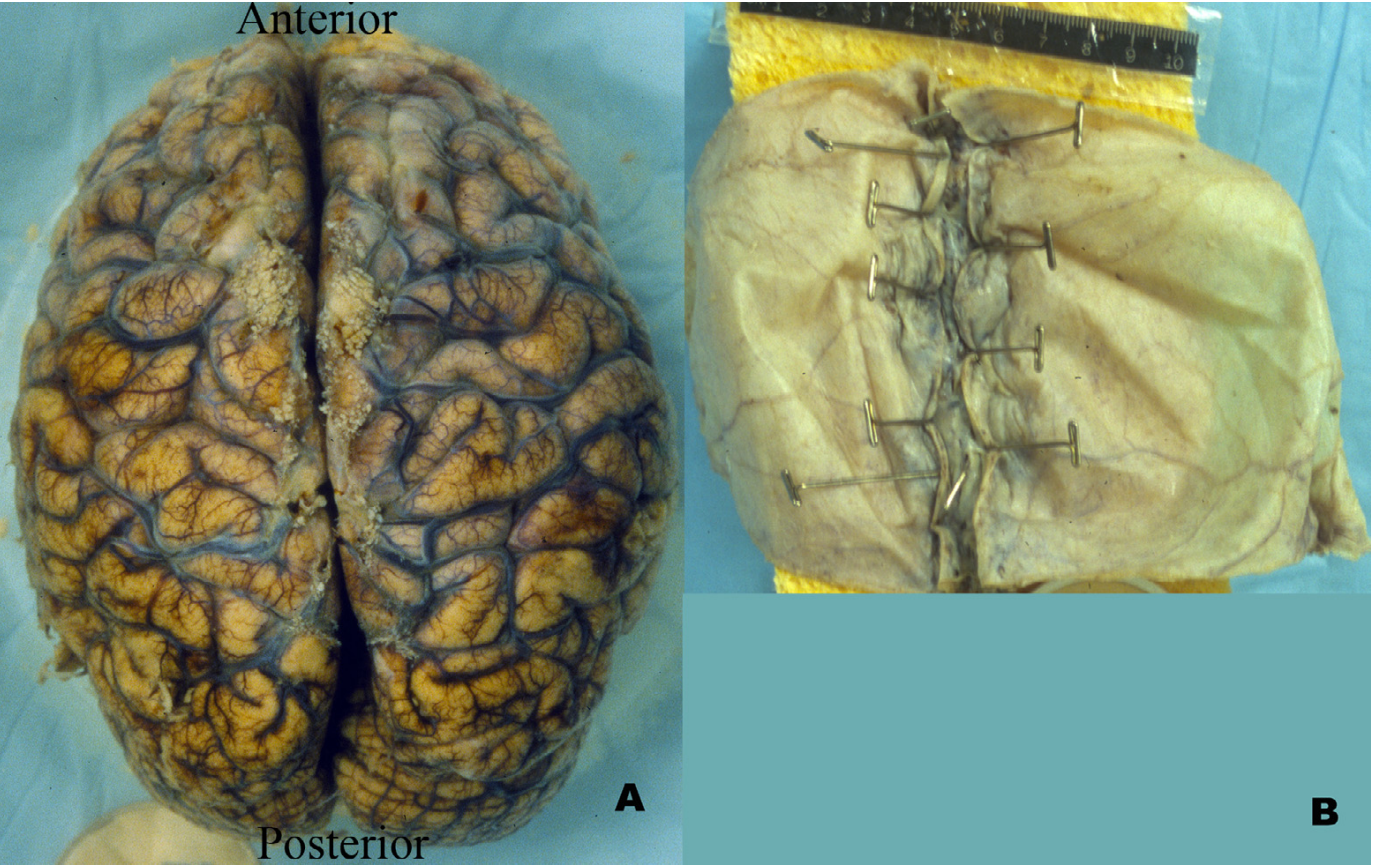
A. Image of human brain with dura removed. The AGs are visible on the brain surface as whitish lobules. B. Image of inside of the dura from a human brain with the superior sagittal sinus splayed open. No AGs are visible in the superior sagittal sinus.

Photographic images were taken with accompanying autopsy reports of inspected, formalin-fixed brains. The brains with accompanying scale and identification were submerged in water to control glare. A 35 mm Nikon FM2 camera with a Micro-NIKKOR 55 mm, 1:2.8 lens was stabilized at a fixed distance. Two images were taken of each brain: the brain with attached scale and identifying tag (Figure 1A, tag and scale not shown); and the superior sagittal sinus splayed open with attached scale and identifying tag (Figure 1B, tag not shown). The film (35 mm 100 ASA Kodak Elite Chrome) was processed and scanned on a Polaroid Sprintscan 4000 35 mm slide scanner. Pixel resolution was approximately 60 μm, suggesting that an individual AG would be approximately 5 pixels in diameter on average [3].

**Figure 2:**
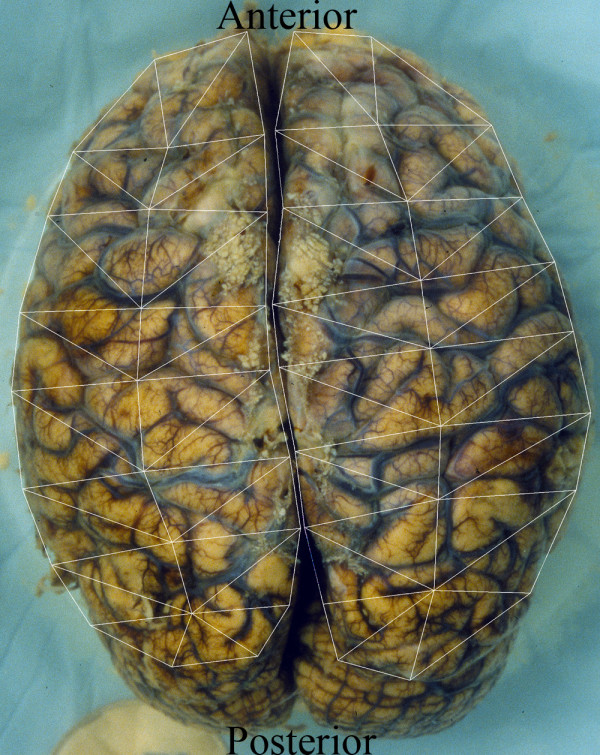
Segmented brain image. This is the same brain as in 1A with marked fiducial points connected.

**Figure 3:**
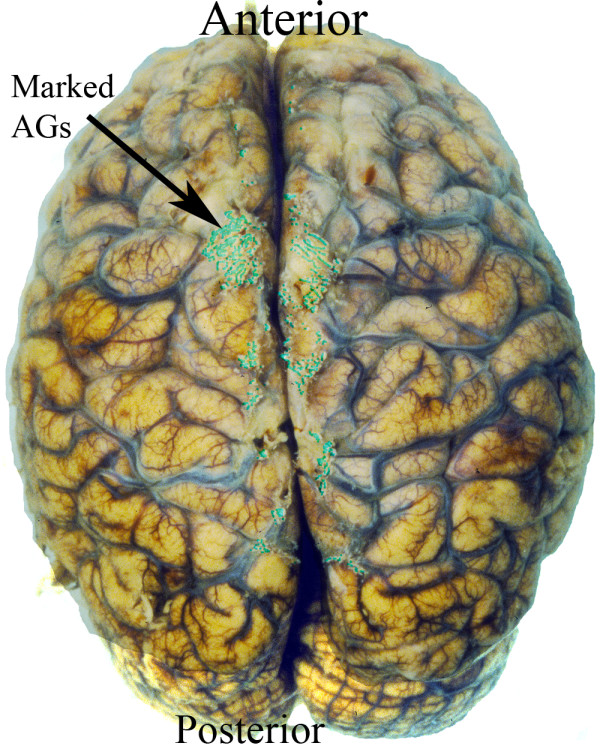
Identified AGs on brain surface using Adobe Photoshop. Positive AG locations are indicated in color.

**Figure 4:**
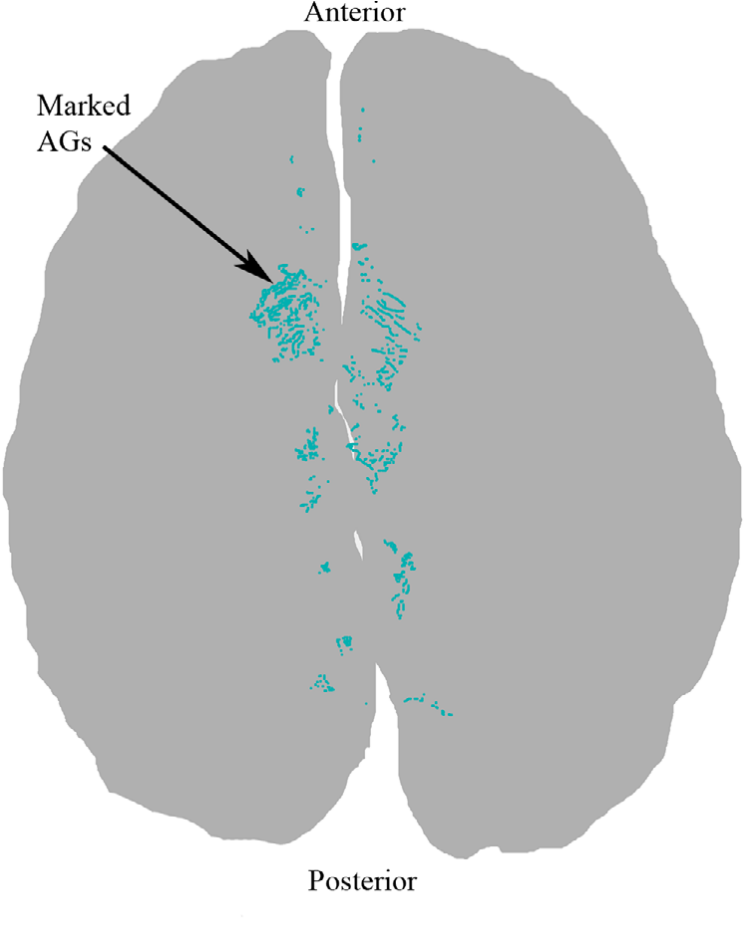
Identified positive AG locations on displayed on the brain hemisphere map.

**Figure 5:**
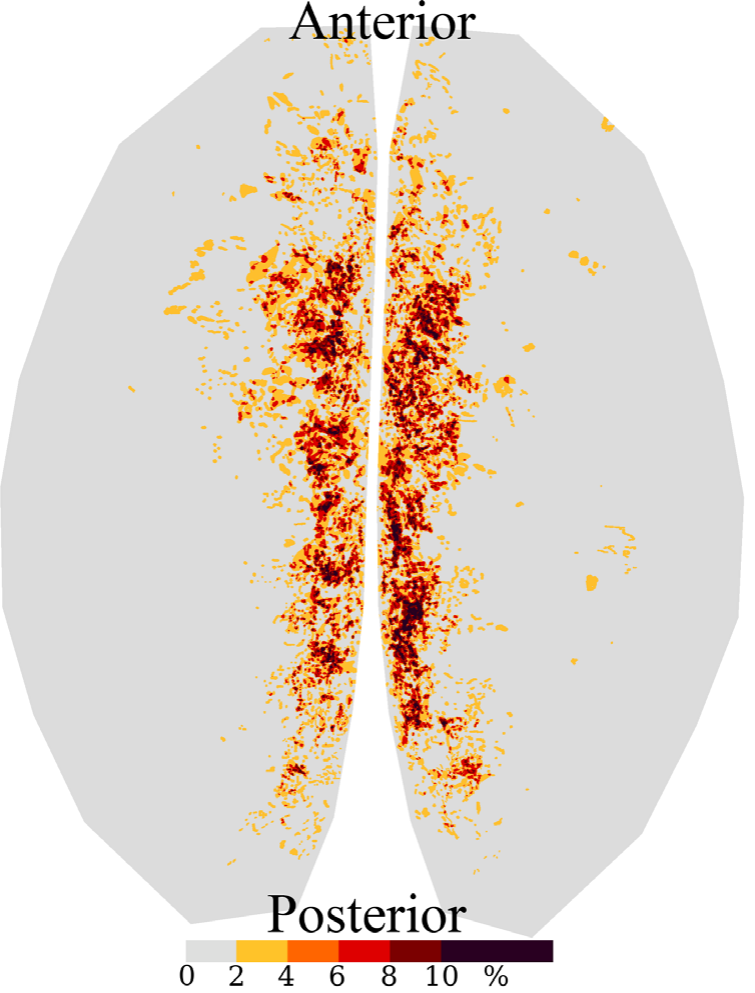
Topographic distribution map using data from 35 brains showing the probability of an AG occurring at each specific pixel on the standard transformed brain template. AGs have a characteristic distribution along the longitudinal fissure. Darkest color in the scale denotes AGs were identified at that pixel in greater than 10% of the analyzed brains. Each progressively lighter color represents a smaller percentage of cases have an AG at that pixel as indicated by the scale.

Briefly, a standard set of 25 fiducial points was identified on each cerebral hemisphere. The fiducial points were equally spaced on each hemisphere to limit spatial variability. A transformation template was created by connecting these 25 fiducial points in each hemisphere. (Figure 2). Each investigator created a segmented image using Adobe Photoshop to manually identify the surface AGs (Figure 3). The identified AGs for one brain are shown on the brain hemisphere image (Figure 4). The same brain was used for Figures 1 through 4. The AG surface area was calculated by counting the number of positive pixels in the segmented image. A topographic distribution map was calculated to assess the spatial distribution of the AGs for all 35 brains in the current data set [7]. An average hemisphere template was calculated by averaging the position of the 25 fiducial points on each hemisphere from all processed brains. Each individual segmented image was spatially transformed to the standard (average) template. The AG surface area was calculated for each hemisphere and for the total brain. To adjust for different brain size, the proportional involvement of AGs was calculated by dividing the AG area by total area. These data were used to create a topographic map depicting the spatial distribution of AGs for all 35 brains analyzed. Each pixel in the topographic map was color coded according to the proportion of AG positive cases at that pixel. A scale was created to quantify the percentage of AGs present at each pixel from the compilation of all analyzed brains after being spatially transformed to the standard template. The scale is a color gradient of 5 colors, where each darker color represents a greater percentage of AGs located at that pixel from the compilation of analyzed brains (Figure 5). The darkest color in the scale denotes that AGs were identified at that pixel position in greater than 10% of the analyzed brains, with progressively lighter colors representing smaller percentages as indicated. A linear regression was used to assess reproducibility between investigators.

## Results

Images have been obtained and analyzed from 35 brains. The average pixel size (linear) was 7.2 ± 0.3 μm (mean, sem), which is small enough to allow visualization of the AGs which have an average cap cell diameter of approximately 300 μm[3]. Images were taken from brain donors of both sexes (21 male, 14 female), of both Caucasian and African-American races (24 Caucasian, 11 African-American), and of ages ranging from 20–85 years (mean 53.1, median 52). Figure 5 shows the AGs for these 35 brains are located primarily along the longitudinal fissure, with the darkest color on the scale representing the greatest relative chance of having an AG present at that location. The regression equation comparing the AG proportion measurements by the two investigators was Investigator2 = 0.00087+1.0025*Investigator1. Regression analysis confirmed reproducibility of AG identification between independent researchers with r^2 ^= 0.97 (data not shown). The average *en face *surface area of AGs was 78.53 mm2 per whole brain. Of the 24 brains that had full intact sinuses, only 4 sinuses contained AGs. Only 1 of the 4 contained granulations in a considerable amount, however, even in this case it was less than 5% of the total AG surface area. With such a small sample it was not possible to determine a pattern of distribution for AGs in the SSS.

## Discussion

As shown in Figure 5, AGs occur with greatest frequency in the parasagittal planes of the superior surface of the human cerebral cortex. The parasagittal planes coincide with the region of the lateral lacunae, suggesting that the AGs occur with greatest frequency in the lateral lacunae, which is in agreement with Le Gros Clark's observations [4]. Our results indicate that AGs are rarely present directly in the SSS, which is potentially in contrast to Le Gros Clark's observation of AGs occurring in the SSS "quite frequently." This potential difference may be attributed to discrepancies in samples between the two studies. Le Gros Clark's study consisted of brains from age 18 months to 4 years and he noted an increase in frequency with age [4]. The present study had a mean subject age of 53.1 years, which could account for some discrepancy in findings. In the current study, it is possible that when the sinus was removed with the dura, AGs were also removed or destroyed. The possibility of this occurring, however, was minimized by the careful dissection and visual inspection employed in the study protocol.

It is important to note the demographic distribution of the population because of the known increased frequency of AGs with age. Gomez *et al*. studied the development of arachnoid villi and granulations in 27 fetuses and newborns aged 26–54 (gestational) weeks, and observed that embryological development of arachnoid villi were "confined to the posterior half of the SSS" [8]. Since the present study did not concentrate on the microvilli, it is possible that there is an abundance of microvilli in the SSS, particularly in the posterior half.

A 1996 study by Fox *et al*. looked at the SSS and parasagittal dura of 20 formalin-fixed human adult cadavers and 15 autopsy specimens and determined that the AGs were located predominantly in the middle third of the walls of the SSS, with decreasing frequency anterior or posterior to this region [5]. As previously mentioned, Le Gros Clark emphasized the distinction between the lateral lacunae and the sagittal sinus, regarding them as two separate anatomic entities, and stating that the lateral lacunae are not mere "diverticula of the sagittal sinus," but rather a complicated "meshwork of veins" [4]. The physical location of the bulk of adult human AGs in the vicinity of the lateral lacunae may give an indication as to functionality. Fox *et al*. noted that the lateral lacunae provide a direct pathway to the venous outflow system, which would allow for the outflow of CSF from the AGs to the venous system [5].

Due to their intracranial location, human AGs can be challenging to study. This fact led Gomez and Potts to study AGs in sheep in an attempt to understand the effects of pressure changes on AGs [9]. They reported that "most [AGs] are located in the posterior third of the superior sagittal sinus", suggesting that the distribution in sheep may be different from that found in the current human study.

Most recent reports of AG frequency are documented in neuroradiological literature. This field has a vested interest in this knowledge since AGs can be large enough to make impressions on the inner table of the skull-cap, which are visible on MRI images and CT scans of the skull and occasionally confused with brain lesions, minor contusions, or other pathology [10]. Accurate documentation of the frequency and appearance of AG impressions are important in this field for correct interpretation of MRI and CT images, and the proper delivery of health care to these patients. The neuroradiology data representing the frequency of very large AGs visible by MRI due to the impression left on the skull-cap, considerably underestimates the true frequency of AGs. Grossman, *et al*. reported in a radiographic study that AGs were located in the superior sagittal sinus, transverse sinus, cavernous sinus, superior petrosal sinus, and straight sinus in decreasing frequency [10]. They also reported that AGs are found with greatest frequency in relation to venous entry sites into the sinus, possibly providing a clue as to their function [10]. Since these frequencies are those of giant AGs, their correlation with the frequency of (non-giant) AGs is speculative.

In addition to characterizing the AG spatial distribution and *en face *surface area, the present study has important potential applications for future studies. We have developed an *in vitro *model of CSF outflow, which uses cells cultured from AG caps seeded onto filter membranes [11]. A flow perfusion system is used to measure the permeability of a confluent layer of these cells at physiological and non-physiological pressures. In order to extrapolate the experimental measure of CSF outflow data to physiologic conditions, an estimate of human AG surface area is needed.

Upon inspection of the macroscopic AGs, the outflow area on each granulation is typically only the apical surface, which is what is projected in an *en face *two-dimensional image of the brain. Therefore, using a two-dimensional representation of a three-dimensional object will have minimal implications in the final analysis. A potential source of underestimation of the total AG surface area is that the imaged AGs are not fixed under pressure. This may reduce the estimated total AG surface area, especially in the diseased state when pressures are increased.

The majority of subjects in this study were over 40 years of age. Studying additional human subjects in the first four decades of life may yield more information about the evolution of AGs, and could provide information about their role in CSF outflow over time. An increase in sample size will allow the investigation of the relationship between surface AGs and independent demographic variables such as age, gender, race, brain weight, body height, body weight, and Body Mass Index. Understanding these relationships will allow for a better understanding of their potential role in CSF egress in health and disease states.

## Conclusion

We have developed a technique to calculate the en-face surface area and topographic distribution maps of human AGs on the superior surface of the cerebral cortex and shown that AGs are localized in a characteristic distribution with regions of high and low probability. The measurements provide data for AG surface area in terms of absolute values as well as proportional area with respect to total brain area. These data will be used as input for our *in vitro *CSF perfusion model and for future studies on demographic variables. Understanding these characteristics of the AGs will allow for a better understanding of their potential role in CSF egress in health and disease states.

## Abbreviations

AG: Arachnoid granulation, CSF: cerebrospinal fluid, SSS: superior sagittal sinus.

## Competing interests

The author(s) declare that they have no competing interests.

## Authors' contributions

DG: conceived of the study, developed the initial imaging procedures, participated in the study design and coordination, and helped to draft and revise the manuscript.

EH: participated in the study data collection design, performed the image analysis, and statistical analysis, and helped write and revise the manuscript.

KK: helped acquire brain images, was the first operator in the image analysis technique, helped write and revise the manuscript.

DH: helped acquire brain images, was the second operator in the image analysis technique validation and helped revise the manuscript.

SEK: contributed clinical significance and helped revise the manuscript.

All authors read and approved the final manuscript.
